# Patient expectations for artificial intelligence transparency and agency: A qualitative study and proportionate framework for clinical artificial intelligence implementation

**DOI:** 10.1371/journal.pdig.0001816

**Published:** 2026-11-04

**Authors:** Guy Stein, Abinav R. Udaiyar, David Bouslov, Howard M. Ross, Charles E. Binkley

**Affiliations:** 1 Hackensack Meridian School of Medicine, Nutley, New Jersey, United States of America; 2 Department of Medicine, Warren Alpert Medical School of Brown University, Providence, Rhode Island, United States of America; 3 Department of Urology, Wake Forest School of Medicine, Winston-Salem, North Carolina, United States of America; 4 Hackensack Meridian Health, Edison, New Jersey, United States of America; 5 Bioethics Program, Columbia University, New York, New York, United States of America; Khomein University of Medical Sciences, IRAN, ISLAMIC REPUBLIC OF

## Abstract

Artificial intelligence (AI) integration in healthcare is expanding rapidly, encompassing applications from ambient clinical scribes to diagnostic support tools. While AI adoption by physicians has recently doubled, research regarding patient expectations for transparency and agency remains limited. Current literature often fails to provide actionable guidance for translating patient perspectives into operational health system frameworks. This study utilizes feedback from Patient and Family Advisory Councils (PFACs) to explore community perspectives on healthcare AI and propose a transparency framework that engenders trust and prioritizes patient-centered care. In this qualitative study, 20 semi-structured interviews and intake surveys of PFAC members across nine hospital sites within a large healthcare network were conducted. Data analysis of interview transcripts employed a hybrid inductive and deductive thematic approach, utilizing Google’s Gemini 2.5 Pro for initial coding and theme generation, followed by rigorous human validation to reach interpretative consensus. Qualitative analysis identified four nested themes: 1) Ethical Prerequisites for AI Integration, 2) Systemic Prerequisites for Trust, 3) Preserving Clinician Oversight, and 4) Strategies for Meaningful Communication. Participants viewed transparency as an ethical “floor” necessary for autonomy and expressed that trust must be earned through evidence of systemic reliability and High Reliability Organization (HRO) principles. Critically, patients expect clinicians to serve as a “bridge” between technology and the patient, acting as the final arbiter of care decisions. These findings provide the foundation for a proportionate framework regarding communication that scales transparency based on clinical risk and the required level of patient agency. The framework categorizes AI engagement into three tiers: disclosure, assent, and consent. By nesting ethical and systemic foundations within actionable steps for clinician oversight and proactive communication, health systems can implement AI in a trust-engendering manner that preserves the sanctity of the patient-provider relationship.

## Author summary

Artificial intelligence is quickly becoming a part of everyday life, including medical care. However, while this new technology is being researched, explored, and implemented for its potential benefits, there has been little integration of patient opinion into current AI systems. In an effort to implement consultative AI, where all stakeholders’ perspectives are taken into account before model implementation, we wanted to understand what patients need to feel comfortable and respected when these technologies are used in their treatment. In order to do this, we spoke directly with patient advisory council members. Our findings show that patient trust is built on transparency and evidence of strong safety standards. The most common theme that was identified by participants was the need for transparency. Participants emphasized that being informed is the starting point that enables them to maintain control of their healthcare. Rooted in the findings from our data, we developed a three-level framework for communication. This system suggests that for low-risk tools, a simple notice is sufficient, while high-stakes decisions require a more detailed discussion and formal patient permission. We hope this work provides a roadmap for healthcare systems to embrace innovation while protecting the vital bond between patients and their doctors.

## 1. Introduction

Artificial-Intelligence (AI)-integrated health care systems have expanded rapidly and include automated responses to patient messages, ambient AI scribes for clinical documentation, diagnostic support tools in radiology, systems that provide intraoperative decision support in surgery, and many others [1–3]. A recent AMA survey found that over the past two years, AI use by physicians in clinical practice has increased by two-fold [4].

While ethical concerns, including confidentiality, data privacy, and bias, have been brought to the forefront of the conversation, relatively less attention has been paid to whether and how patients want to be informed about the AI systems used in their care and the degree of agency they expect to have [5]. In response, many studies have been carried out to elicit patient perspectives on AI [6–8]. However, there has been a lack of research aimed at providing more actionable guidance on how to translate empirical insights into frameworks that can be operationalized by health systems to promote patient transparency and trust in AI-enabled systems [7].

Consultative AI is a less explored approach that requires stakeholders to have a meaningful say in the AI systems that will impact them [9]. When it comes to healthcare, arguably the most significant stakeholders are the patients. Recent studies have demonstrated that patients want to be included in the AI development and implementation process [6,8,10]. Patient engagement has been shown to improve certain healthcare outcomes, such as hospital admissions, and the quality of health services [11].

The manner in which AI is integrated into the workflow and governed will have major implications for patient outcomes going forward. Therefore, it is crucial for patients to have a high level of engagement within the realm of AI implementation. Patient and Family Advisory Councils (PFACs) are composed of patients, family members, and community representatives. They provide feedback and input to health systems and hospital leadership on matters related to patient-centered care and advocacy.

The aim of this study is to explore community perspectives on healthcare AI, utilizing PFAC members across a large healthcare network, and to propose a transparency framework for AI implementation that will engender trust from patients. Our goal is to develop actionable insights from subjects and propose strategies to enhance patient trust in AI systems.

## 2. Methods

### 2.1. Ethics statement

This study was reviewed and approved by the Hackensack Meridian Health (HMH) Institutional Review Board (IRB ID Pro2024-0296; expedited review; approved November 18, 2024). The IRB determined that the study posed no greater than minimal risk and approved a waiver of documentation of informed consent. Participants received an IRB-approved information sheet describing the study, and consent was implied by completion of the intake survey and interview; written consent was not obtained.

### 2.2. Study design

This study employed a qualitative design utilizing semi-structured interviews and intake surveys to explore patient and community perspectives on the implementation of AI in healthcare settings. The study was conducted virtually.

### 2.3. Study population and recruitment

PFACs affiliated with nine hospital sites within the HMH network. Individuals were initially recruited via virtual presentations at monthly PFAC meetings. Those who expressed interest were then emailed an informational sheet containing all the details of the study, along with a link to a REDCap intake survey.

### 2.4. Data collection

Participants first completed an intake survey, administered through the REDCap platform, which collected basic demographic information and used Likert-scale questions (S1 Text) to assess general attitudes toward AI in healthcare.

After completion of the intake, individual semi-structured interviews were conducted. Interviews were held via Google Meet by one of two trained interviewers and lasted approximately 20 minutes. Each interview incorporated both vignette-based scenarios and open-ended questions (S2 Text) designed to elicit nuanced perspectives on the ethical, practical, and interpersonal aspects of AI use in healthcare. All sessions were recorded using Google Meet’s screen recording feature with automated closed captioning enabled to facilitate transcript generation. Following the interviews, transcripts were reviewed and edited for transcription accuracy, including correction of spelling errors and clarification. All personally identifying information was removed to ensure participant confidentiality.

### 2.5. Data analysis

A qualitative thematic analysis was conducted on the de-identified interview transcripts using a hybrid inductive and deductive approach adapted from Braun & Clarke for use with a large language model (LLM) [12–14]. All LLM-assisted analysis was performed using Google’s Gemini 2.5 Pro Preview (gemini-2.5-pro-preview-06-05), accessed via Google AI Studio. Top-p was left at the model default of 0.95 and top-k at the model default of 64. Temperature was varied across the three analysis modules; verbatim prompts for each module are provided in S3 Text.

The LLM-assisted analytic pipeline comprised three modules; a fourth module supporting illustrative-quote selection is described in the S3 Text *Module 1 (Open Coding; temperature 0.2):* each participant response was passed to the LLM with a structured prompt to identify and code distinct points, arguments, conditions, or emotions. *Module 2 (Code Consolidation; temperature 0.3):* the full set of codes from Module 1 was passed to the LLM to merge conceptually similar codes and produce a draft codebook of 16 consolidated codes. *Module 3 (Theme Generation; temperature 0.6):* the refined codebook was passed to the LLM to propose overarching themes; Module 3 was run multiple times to surface alternative thematic structures before researcher selection. Outputs from each module were reviewed by the research team through iterative discussion to reach interpretative consensus, consistent with reflexive thematic analysis as articulated by Braun & Clarke [15,16].

Reflexive thematic analysis does not endorse inter-rater reliability statistics as quality criteria [15,16]; nevertheless, a retrospective IRR sub-study was conducted for methodological transparency. Three team members independently coded a random subsample of three transcripts (Interviews 1, 4, 9; 15% of corpus; random seed 42) using the finalized 16-code codebook, blinded to the LLM’s outputs. Cohen’s κ at the code-presence level was computed pairwise. Mean human-vs-LLM κ was 0.337 (range 0.269–0.437; 95% CI [0.045, 0.522]); mean human-vs-human κ was 0.378 (range 0.231–0.497), both indicating fair agreement (Landis & Koch 1977). The equivalence of human-vs-LLM and human-vs-human agreement suggests the LLM operated at a level of consistency comparable to that of an independent human coder on this subsample. Per-pair κ values are reported in S3 Text.

## 3. Results

### 3.1. Participant characteristics and survey responses

A total of 21 members of Patient and Family Advisory Councils (PFACs) completed the initial intake survey. Of these, 20 participants (95%) completed the semi-structured interview. The sample was predominantly female (62%, n = 13) and White/non-Hispanic (95%, n = 20). Participants were highly educated, with 17 (81%) holding at least a bachelor’s degree, including eight with master’s or professional qualifications and two with doctorates.

### 3.2. Baseline attitudes toward AI (Intake Survey)

Mean Likert scores (1 = strongly disagree, 5 = strongly agree) from the 20 interviewed participants appear in Table 1 (S2 Data). Respondents agreed that “AI has a role in health-care” (M = 4.29) and that “all patients should be informed about how AI is used” (M = 4.48). However, they were neutral on the need for an opt-out option (M = 3.33) and on the adequacy of current AI safeguards (M = 3.14). Participants reported low-to-neutral understanding of how AI models are developed (M = 2.71) and how they work (M = 3.00). Crucially, they strongly rejected allowing AI to make treatment decisions autonomously (M = 1.38).

**Table 1:** Average scores from Likert Questionnaire from intake survey (n = 21).

Question	Average Score (n = 21)	Average Likert Correlation
I understand how artificial intelligence models are developed.	**2.71**	*Neutral*
I understand how artificial intelligence models work.	**3**	*Neutral*
Artificial intelligence has a role to play in the healthcare field.	**4.29**	*Agree*
All patients should be informed about how artificial intelligence is used in their healthcare.	**4.48**	*Agree*
Patients should have the option to opt out of artificial intelligence use in their healthcare.	**3.33**	*Neutral*
AI models should be given sole responsibility in making decisions on a patient’s treatment plan.	**1.38**	*Strongly Disagree*
The way artificial intelligence programs are utilized in healthcare today is acceptable in taking into account patient’s rights.	**3.14**	*Neutral*

Scale: Strongly Disagree (1), Disagree (2), Neutral (3), Agree (4), Strongly Agree (5)

### 3.3. Qualitative findings

The 20 interview transcripts generated 1,242 initial codes. After iterative consolidation, 16 consolidated codes remained, organized into four overarching themes (Fig 1). The themes, in order of prevalence, were: Ethical Prerequisites for AI integration (n = 46 code mentions), Preserving Clinician Oversight (n = 45), Systemic Prerequisites for Trust (n = 30), and Strategies for Meaningful Communication (n = 25).

**Fig 1:**
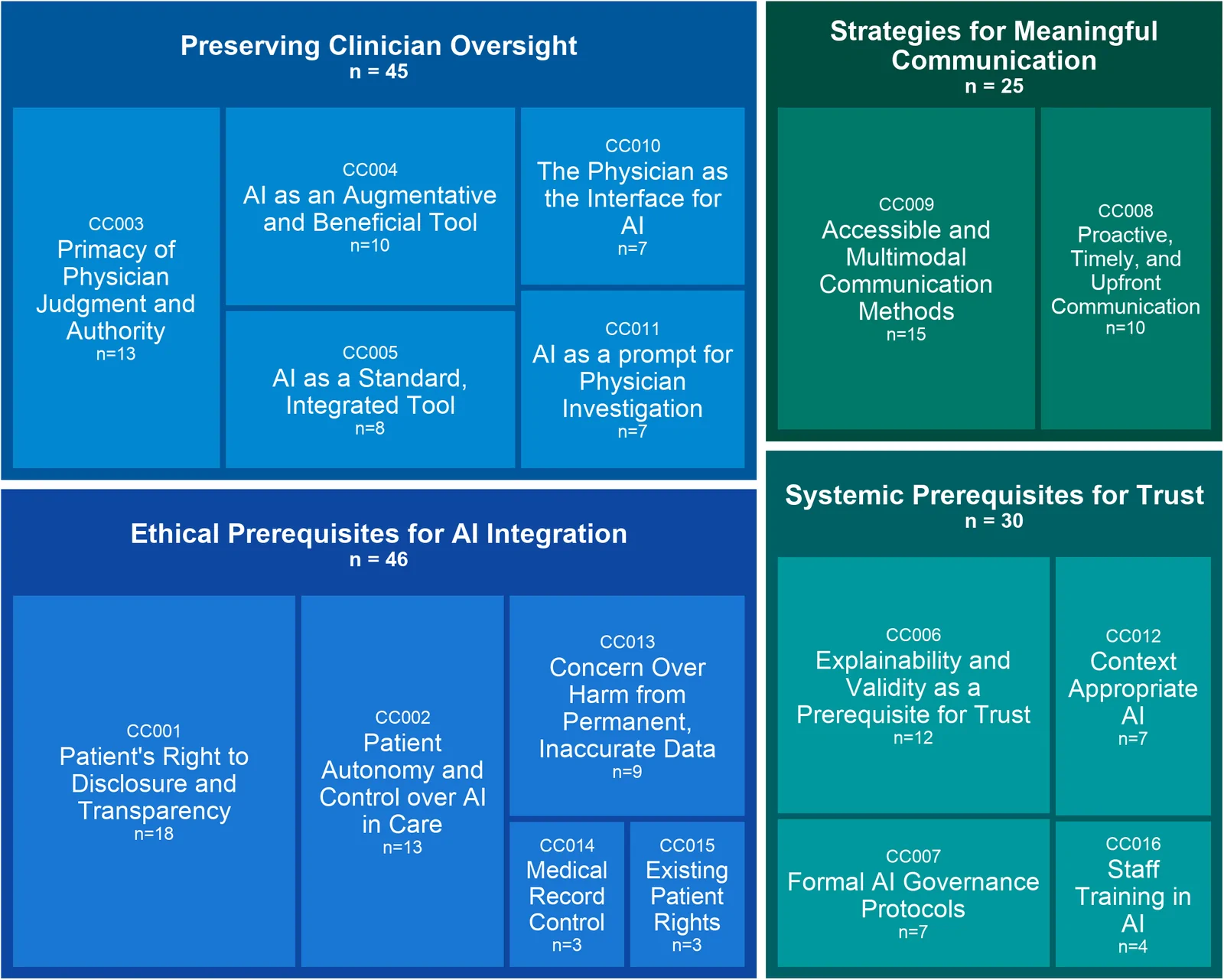
Treemap of four major themes from the AI-in-healthcare interview study. The *Ethical Prerequisites for AI integration* theme (n = 46) occupies the largest area and contains five codes; “Patient’s Right to Disclosure and Transparency” (n = 18) is its largest block. *Preserving Clinician Oversight* (n = 45) is almost as large, led by “Primacy of Physician Judgment” (n = 13). *Systemic Prerequisites for Trust* (n = 30) clusters explainability, governance, and context-appropriate use, while *Strategies for Meaningful Communication* (n = 25) comprises two codes on multimodal, proactive communication. Each rectangle’s area reflects participant frequency.

The first theme, and the most commonly mentioned, is the Ethical Prerequisites for AI Integration. This theme captures the fundamental, non-negotiable principles that participants believe must be preserved in the age of AI. At its core is the conviction that technology must not erode the patient-provider dynamic and prioritize care to be patient-centered. The most mentioned code within this theme and throughout all interviews revolves around disclosure and transparency of information, which participants viewed as foundational to their autonomy and ability to exercise agency over AI use in their care. Additionally, participants expressed a similar desire to control their medical records, citing concerns of permanent harm done by inaccurate data. Despite not having formal knowledge of the systems that protect patients in healthcare, some participants still expressed a clear desire to take action, drawing on the frameworks they already trusted. These ideas for putting patient-centered care into practice lead us into the next major theme: the Systemic Prerequisites for Trust.

The second major theme focuses on the structural and procedural safeguards that participants believe must be in place for healthcare AI to earn their trust. Participants consistently emphasized that trust is not inherent—it must be earned through systems that ensure clarity, oversight, and responsible use. A central sentiment within this theme is the ability to explain an AI model and validate the reasoning behind its use. Building on this, participants called for clear oversight and standardized protocols to guide how AI is implemented, how disagreements between AI and clinicians are handled, and how patient consent or refusal for AI-enabled care systems is obtained. Participants also stressed that AI should be used in an appropriate context. Underlying all these expectations is the belief that healthcare professionals utilizing AI in a clinical setting must be properly trained, with competence viewed as essential for safe and effective use. Taken together, these elements reflect the macro-level mechanisms associated with operationalizing patient-centered care. The subsequent theme examines the micro-level relationship participants outlined between themselves, clinicians, and AI.

The third major theme describes the ideal human-AI relationship from the patient’s perspective. Participants consistently reinforced that their trust was ultimately placed in their ‘human’ clinician, and their acceptance of AI is conditional upon the clear involvement of their trusted clinician. Participants welcomed AI as an augmentative and beneficial tool that supports and strengthens the clinician’s capabilities, but insisted that the physician be the final decision maker in care. Some participants even normalized AI as another tool in the modern clinic. Critically, participants expect the clinician to serve as the primary communicator when explaining the technology’s role in the patient’s care or providing personal endorsement regarding AI. These expectations regarding the clinician’s central role in mediating AI during the clinical encounter naturally connect to the final theme, in which participants extend this reliance on the clinician as the primary human interface for AI to their communication preferences about the technology as a whole. Notably, the expectation that the clinician serve as the primary interpreter and communicator of AI emerged within this and the subsequent theme, forming a direct bridge between the two and focusing on participants’ specific communication expectations regarding AI.

The fourth major theme outlines the communication practices participants expect when AI is incorporated into their care, further extending the importance they place on the clinician’s role as primary communicator. Across interviews, participants emphasized the importance of proactive, timely, and upfront communication, expressing a preference to be informed about AI use well before moments of stress or clinical urgency. Participants also highlighted the need for accessible and multimodal communication methods, noting that clear language, written materials, and visual aids are essential for meaningful understanding. Collectively, this theme demonstrates that participants place equal importance on how information about AI is communicated and who communicates it, reinforcing the clinician’s central role in shaping patient understanding and trust.

Taken together, the themes demonstrate an interdependent pattern, with each theme emerging from and supported by elements identified elsewhere in the analysis. This progression reflects a coherent patient perspective centered on protected rights, trustworthy systems, clinician-led decision-making, and clear communication about AI.

## 4. Discussion

Analysis of the interview data yielded four central themes regarding AI implementation: 1) Ethical Prerequisites for AI Integration, 2) Systemic Prerequisites for Trust, 3) Preserving Clinician Oversight, and 4) Strategies for Meaningful Communication. Transparency and agency emerged as the most significant concerns for participants. This suggests that for participants, transparency is not merely a technical requirement of AI, but an ethical “floor” that enables the exercise of agency. Participants expressed a clear concern that a lack of transparency would lead to a loss of agency over their own health, signaling that any successful AI integration must be built on the same trust-based foundations that govern the traditional patient-provider relationship.

The first theme, *Ethical Prerequisites for AI Integration*, establishes the non-negotiable principles patients expect as AI enters the clinical setting. Beyond simple notification, patients viewed disclosure and transparency as the mechanisms that protect their right to make decisions. This was particularly evident in discussions regarding medical record accuracy; participants viewed their data not just as information, but as a representation of their personhood that requires protection. These findings echo the bioethical principle of respect for autonomy, demonstrating that transparency is not merely what patients prefer but the mechanism that makes autonomous decision-making in healthcare possible [17]. Overall, patients do not view AI as a neutral tool, but as an intervention that requires explicit ethical alignment with patient-centered care. The first theme is unique in establishing the ethical foundations upon which all subsequent themes will stand.

The second theme, *Systemic Prerequisites for Trust*, moves from abstract ethical principles to the macro-level safeguards required for institutional trust. Our findings indicate that participants do not inherently grant trust to AI; rather, trust must be earned through evidence of systemic reliability. This includes standardized protocols, rigorous validation of AI models, and staff training. This pattern parallels findings from the public-health response to COVID-19, in which systemic trust in healthcare infrastructure, rather than confidence in any single tool, was the primary determinant of public adherence to novel interventions. AI systems introduced into low-trust institutional environments are unlikely to be accepted by patients regardless of the technology’s intrinsic reliability [18]. Participants emphasized that for an AI system to be trustworthy, the humans operating it must demonstrate competence. This mirrors the principles of High Reliability Organizations (HROs) defined by the Agency for Healthcare Research and Quality, which are characterized by a collective commitment to a safety-focused culture and standardized excellence [19]. By demanding such rigorous systemic oversight, patients are essentially calling for AI to be integrated into the same culture of safety and excellence that defines the most reliable healthcare networks.

Regarding *Preservation of Clinician Oversight,* our data show that participants do not view AI as a replacement for human judgment, but as an augmentative tool that must remain under human supervision. At the heart of this theme is defining the new three-way AI-clinician-patient relationship. Traditionally, the clinician-patient relationship has been the basic dynamic in which modern healthcare is delivered and shared decision-making occurs. Looking ahead to the age of AI, the AI-clinician-patient relationship will need to meet those same standards, with participants viewing clinicians as the “bridge” between humans and technology. As a bridge, clinicians are entrusted by their patients to interpret AI outputs, validate recommendations, and serve as the final arbiter of care decisions. This positioning of the clinician as a trusted intermediary is consistent with implicit leadership theories (ILTs), which describe how patients carry pre-formulated expectations of clinicians as authoritative interpreters and may use these expectations to determine whether to extend trust to new technologies introduced into clinical encounters [20].

This trust also has a practical implication for how clinician-AI disagreement should be handled: because patients have already delegated final judgment to their physician, a clinician overriding or discounting an AI recommendation is not a transparency failure requiring disclosure; it is precisely the oversight patients expect. Just as clinicians routinely adjudicate between conflicting data sources (discordant imaging and laboratory results, contradictory specialist opinions, etc.) without itemized explanation, AI output is best understood as another input absorbed into existing clinical judgment rather than as a parallel decision-making authority that must be publicly reconciled. From our data, it was clear that transparency between clinicians and their patients regarding AI involvement in their care should be known; however, the intricacies of how clinicians use AI input to make final clinical decisions, for example, disagreement with AI output and the clinician, do not require disclosure. Importantly, our findings suggest that patients do not experience an inherent tension between their desire for transparency and the necessity of clinician oversight. Rather, patients inherently harmonize these principles by delegating operational decision-making to the provider, expecting overarching transparency regarding AI’s presence in their care while trusting the clinician’s judgment to determine when and how specific algorithmic inputs are relevant to discuss.

Finally, *Strategies for Meaningful Communication* highlights that “how” and “when” information is shared is as important as “what” is shared. Furthermore, it emphasizes the role of clear communication in engendering trust in patient care. Patients expressed a preference for upfront, proactive, multimodal communication delivered well before moments of clinical urgency to give patients enough time to process the implications of AI use. In addition to characterizing each theme, we explored the relationships between them. In doing so, we found that the four themes relate to one another in a nested fashion. The ethical principles (Theme 1) inform the systemic requirements (Theme 2), which in turn empower the clinician to maintain oversight (Theme 3) and communicate effectively (Theme 4). While themes one and two provide the logical and macro-level building blocks for patient trust, themes three and four represent actionable steps that can be taken for AI implementation in a trust-engendering manner (Fig 2).

**Fig 2:**
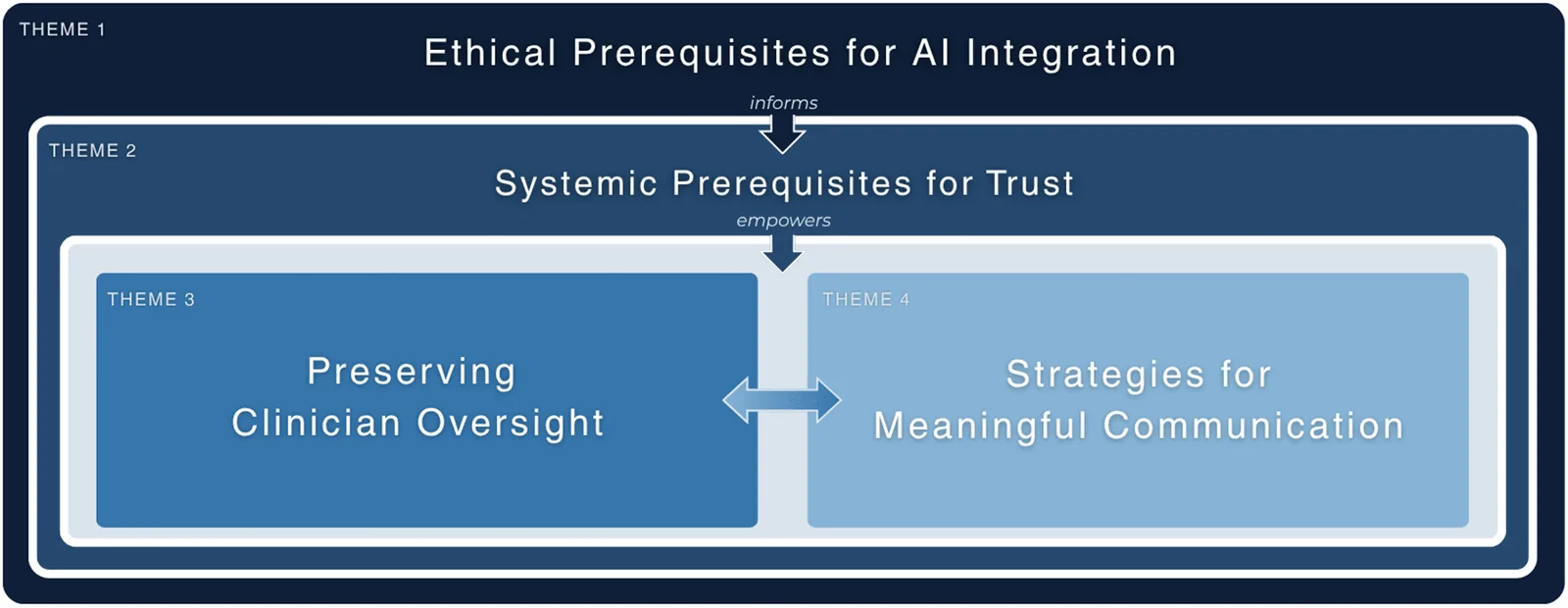
Visual representation of “nesting” relationship between themes. The first theme *Ethical Prerequisites for AI Integration* encompasses and provides the theoretical backbone for the other three themes. Nested within this first theme is the second theme *Systemic Prerequisites for Trust* which covers the system level infrastructure necessary for trust. Within the second theme reside themes three (*Preserving Clinician Oversight*) and four (*Strategies for Meaningful Communication*) which operationalize the foundations laid out by the previous themes.

A proportionate and escalating framework for patient transparency, informed by this study’s findings, considers the degree of both information and agency that is appropriate for a given clinical AI solution (Table 2). The framework that we propose parses information and agency into three distinct categories: disclosure, assent, and informed consent. These three categories both reflect current clinical practice and what participants themselves described (Table 2). For routine applications, participants were comfortable with a simple notification: *‘ultimately I think one disclosure is fine per setting.’* But as AI involvement increased, so did expectations: *‘I want somebody to tell it to me in patient terms.’* And for decisions affecting treatment, participants were unequivocal: *‘the patient needs to be able to make the final decision’.* This tiered approach directly responds to participants’ expectations: it allows for clinician involvement at critical decision points (Theme 3), ensures information is delivered appropriately (Theme 4), while operationalizing the ethical and systemic foundations patients identified (Themes 1 and 2). The following description of the framework outlines a potential structure for a clinically practical approach to implementing AI in healthcare that emerged from patients’ perspectives and keeps them central.

**Table 2:** Defining disclosure, assent, and informed consent as applied to AI-enabled systems in healthcare.

	Disclosure	Assent	Informed Consent
**Information Provided**	Written notification that AI-enabled systems are being used(website, general disclosures, public-facing patient materials)	Verbal or written notification that AI-enabled systems will be used if assent is given for their use	Risks, benefits, and alternatives to AI-enabled tools are discussed
**Agency**	No act of volition is required	Assent is required	Consent is required
**Current Practice**	Routine diagnosis and treatment	Physical exam, IV placement	Surgery, invasive procedures
**Application to AI**	Low-risk AI-enabled systems, characterized by high reliability, low risk of automation bias, and a high level of data protection/sensitive data	Intermediate risk AI-enabled systems characterized by moderate reliability, risk of automation bias or reduction in clinician oversight, and data sensitivity	High-risk AI-enabled systems characterized by lower reliability, autonomous function or nominal autonomy due to high risk of automation bias, and highly sensitive data

Each communication tier is compared across four dimensions: information provided, patient agency, analogous current clinical practice, and proposed application to AI. This tiered structure reflects participant consensus that the appropriate level of patient communication should be proportionate to the clinical risk posed by the AI-enabled system.

The proportionate degrees of information and agency are determined by taking into account the value patients may place on knowing that the model is incorporated into their care, whether patient preferences about the model’s use in their care can be accommodated, and the risk of harm related to acceptance or refusal of AI-enabled care. Risk in AI-enabled systems is multidimensional, encompassing factors such as the degree of model autonomy, the extent to which the system departs from established standards of care, whether the AI is patient-facing, and the direct clinical risk introduced by the system within its care context [21]. Rather than prescribing a fixed risk typology, this framework is intentionally designed to be generalizable, recognizing that health systems must evaluate these dimensions within their own governance structures and local ecosystems. Disclosure would be adequate for low-risk AI-enabled systems, which is true of most care systems. Assent is proportional for intermediate-risk AI-enabled systems, or when refusal of the AI-enabled system does not increase the risk of harm. Informed consent/refusal is proportionate for high-risk AI-enabled systems and for intermediate-risk models, for which refusal increases the risk of harm (Fig 3).

**Fig 3:**
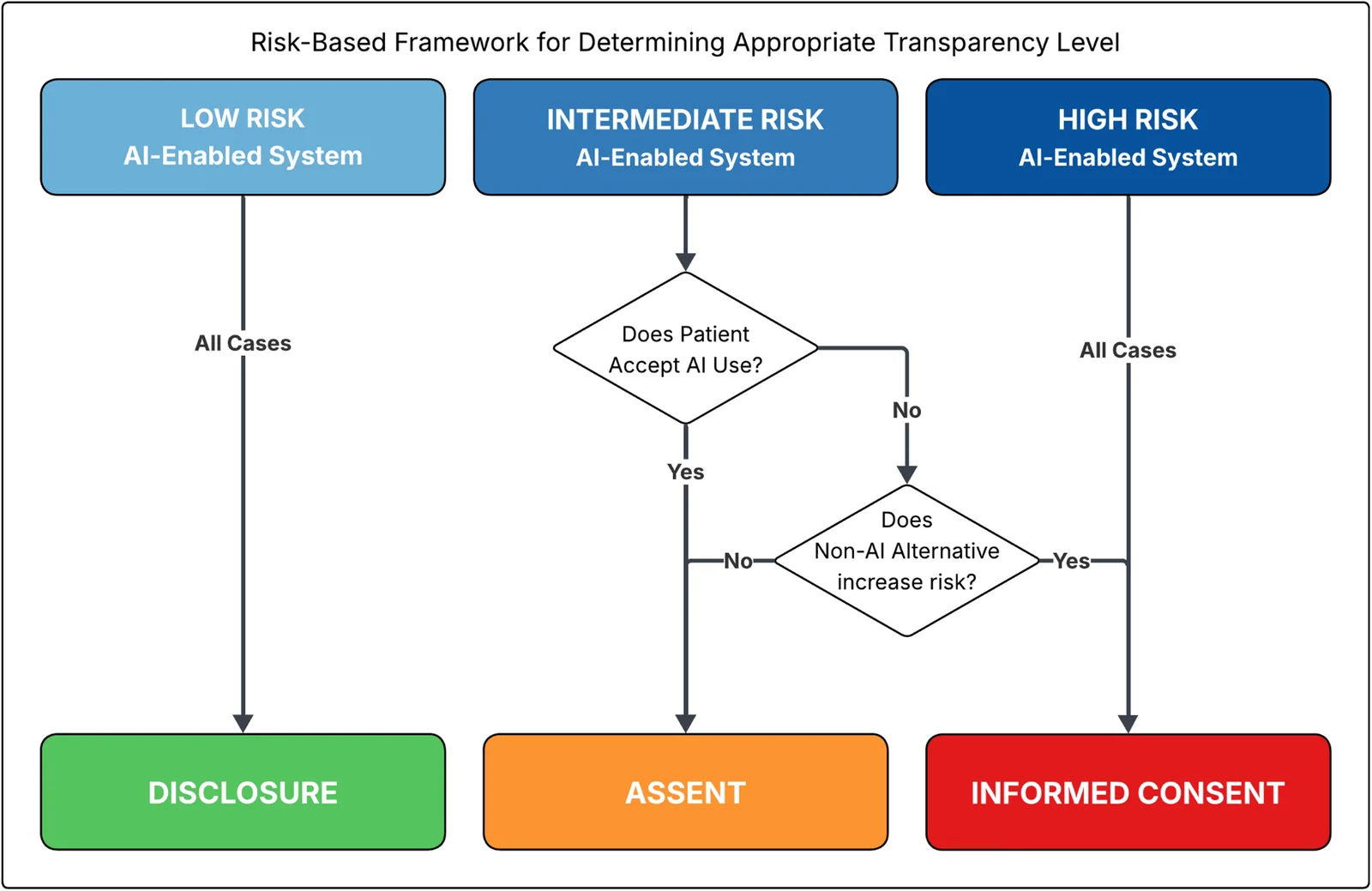
Risk-based framework for determining appropriate transparency level. This figure represents a framework that stratifies communication regarding AI in patient care based on the risk of the given AI-enabled system. Low risk systems, which represent the majority of cases, would result in disclosure. Intermediate risk systems can be differentiated from high risk systems by evaluating patient acceptance of AI use and the risk introduced by a non-AI alternative.

While the framework’s three tiers share structural similarities with established clinical risk stratification, the attributes governing tier assignment are specific to AI-enabled systems and are not fully captured by conventional procedural risk frameworks. Several AI-specific properties can independently elevate a system’s tier designation. Non-explainability, or the inability of a clinician to reconstruct or communicate the reasoning behind an AI recommendation to at least a minimal degree, undermines the informational preconditions for meaningful consent. However, a core component of AI models is the ability to make calculations that are not readily explainable to a human observer. Attempting to force greater interpretability onto a model can degrade its accuracy and clinical efficacy [22,23]. Reliability, or the extent of confidence that one can ascribe to an AI output within a specific context, is therefore a more appropriate measure for AI models. In medicine, this form of practice is already being followed. For example, most physicians cannot explain the exact workings of a CT scan, but still rely on its output to make clinical decisions because of its high reliability. In this logic, AI systems that demonstrate high reliability even in settings of elevated clinical risk may still require only assent, whereas low-reliability systems would require consent due to the higher potential for error and patient harm.

Clinician oversight and system autonomy are other risk identifiers for health AI. As participants in our study mentioned, many had concerns about clinician oversight of AI models and about how autonomously functioning models may impact their care. While a fully autonomous model involving clinical care may automatically require consent. Additionally, systems that may be prone to automation bias, which may lead to a functional reduction in clinician oversight [24]. Therefore, systems that are truly autonomous or highly prone to automation bias should require a consent-level obligation, as they have a higher potential to override the clinician oversight highlighted in theme 3 of our data.

Proprietary data retention and restricted patient access to AI-generated outputs introduce ongoing privacy risks that can elevate a nominally low-risk tool to assent-level when patients’ ability to correct or contest AI-generated data is limited. As our data demonstrates, participants highly valued their privacy and their ability to protest components of the medical record. Therefore, systems that expose greater amounts of confidential data (i.e., voice recording) or medical records would be considered higher risk.

Together, these attributes constitute the AI-specific “conceptual machinery” that differentiates this framework from generic risk stratification. When determining the appropriate level of patient engagement, it is not only the magnitude of clinical risk but the explainability, controllability, and data governance properties of the model of interest.

In line with what others have reported, in this framework, not every AI application requires the full exercise of informed consent/refusal, which entails both detailed consideration of risks, benefits, and alternatives and the volitional act of consent or refusal [25,26]. Requiring informed consent for every AI application is neither justified nor feasible [25,27]. Some AI applications pose so little risk of harm that such consideration is unnecessary and overly burdensome, and disclosure is adequate. However, informed consent/refusal is required both for high-risk AI-enabled systems and for the refusal of intermediate-risk AI-enabled systems if refusal increases the risk of harm.

Several features of this framework differ from those proposed by others and are worth highlighting. First, the framework considers the inherent value that patients may place on knowing that AI is being used in their care. Disclosure respects individual patients’ informational preferences and recognizes that some patients will value knowing that AI is being used in their care, even if they can not refuse. The framework does not assume that every patient will find value in knowing that AI is being used in their care, but for those who do, the information is available through disclosure. Similarly, disclosure respects the informational preferences of patients for whom knowledge of AI in their care is unimportant and of those who may judge for themselves that such knowledge is more harmful than beneficial.

Blanket rejection of disclosure because, in some instances, it may be harmful, or because disclosure is determined not to be in the patient’s best interests, risks an overly paternalistic framework and may limit patients’ agency [28]. Disclosure that AI is being used in a patient’s care has also been discounted based on the argument that AI for decision support is similar to consulting with a colleague or using standard, non-AI-enabled clinical tools [25,29]. As the argument goes, because clinicians do not customarily disclose these modes of decision support, neither should they be expected to disclose AI-enabled decision support. Apart from the substantive ways AI tools differ: their lack of explainability and inconsistent proof of clinical benefit, that they typically prompt an action rather than provide information, and that they are almost always pushed out to clinicians rather than being requested, patients have clearly indicated that they want to know when AI is being used in their care, both in the current study and in other studies [30]. Patients’ stated preference for knowing when AI is used in their care is a stronger argument in favor of disclosure than the hypothetical concerns to the contrary.

Another feature of the proposed framework is its recognition of a middle ground between simple disclosure for low-risk AI-enabled systems and the process of informed consent/refusal for high-risk systems. Within this framework, assent requires more than simple disclosure but less than a full consideration of risks, benefits, and alternatives. It is proportionate for intermediate risk AI-enabled systems over which patients have agency to accept or refuse. Assent would be appropriately applied to ambient AI scribes, as there is an intermediate risk of harm, but a full informed consent discussion is not warranted. This is because passive audio capture in sensitive clinical encounters introduces data privacy vulnerabilities, and transcription inaccuracies can result in documentation errors with downstream clinical consequences. Our data showed that participants independently raised concerns about medical record accuracy and the permanence of erroneous data, lending direct empirical support to this classification. Furthermore, applying Rose & Shapiro’s criteria, ambient scribes are patient-facing, present during the clinical encounter, and represent a meaningful departure from traditional documentation practice [21]. These factors would collectively demonstrate an intermediate level of risk rather than a high one and support placement in the assent tier rather than simple disclosure.

A third feature of this framework is that it recognizes patient preferences to accept or refuse intermediate and high-risk AI-enabled care systems, even if such refusal adds additional risk to their care. Current clinical and ethical practice recognizes patients’ ability to refuse care even if doing so increases their risk. For instance, if a patient refuses to allow the use of an intermediate-risk ambient AI scribe, the clinician would complete clinical documentation without the AI scribe’s assistance, without increasing risk. However, if a patient refuses an intermediate-risk AI-enabled mental health chatbot, which has been clinically proven to improve the effectiveness of psychotherapy for treating major depressive disorder, the patient may be at greater risk than had they accepted the chatbot.

Finally, when considering risk of harm, this framework shifts focus from the intrinsic risk of the AI model itself and looks holistically at the risk of an AI-enabled system of care, recognizing that AI models exist within care delivery systems and that the model may increase or decrease the overall risks of the system into which it is being deployed [31]. The risks and benefits of an AI-enabled system, rather than a model in isolation, are more accurate reflections of real-world realities and are more easily captured in current safety and quality measures. For example, an AI-enabled chatbot itself may pose risks related to over-reliance, data privacy, and humanization. However, when it is embedded into a care system, overseen by a psychotherapist, and subject to bioethical norms, its risks are lessened. Conversely, a model that predicts patients’ 3-month mortality rates may pose little risk in itself. However, when it is used in an acute care setting, the prediction may be misused as a marker of medical futility, leading to the withholding of beneficial treatments based on a patient’s predicted high risk of dying. In adopting this framework and determining whether an AI-enabled system is high-, intermediate-, or low-risk, health systems and their AI governance structures will need to carefully consider their own risk tolerance, how individual AI-enabled care systems function, and the unique risks they may pose within their local ecosystem.

### 4.1. Limitations

While this study provides a patient-centered framework for clinical AI implementation, it comes with its own limitations. The participant cohort was predominantly female, caucasian, and highly educated, which makes this sample unlikely to be generalizable to the general public. However, this skew was an expected limitation in our study design, as we intentionally sought out members of PFACs who serve as “key informants” that may possess unique insights given their experience in patient advocacy within a healthcare system. Engaging with this specialized population allowed us to establish a robust baseline for an ideal transparency framework. Nonetheless, we recognize that perceptions of AI, data privacy, and trust in medical institutions are heavily influenced by cultural, socioeconomic, and geographic factors. In the spirit of consultative AI, future studies are planned to build on this baseline framework and evaluate it in a broader, more generalizable patient population. This will ensure the final operational frameworks are adapted to protect and serve all populations. A further limitation concerns the nature of stakeholder engagement in this work. PFAC members were consulted to elicit their opinions, but they did not influence the design, deployment criteria, or governance of any AI model. This study should therefore be understood as a consultative rather than a fully participatory effort, and future work should engage stakeholders more directly in model development, deployment decisions, and oversight.

## 5. Conclusion

Participants in this study emphasized the need for transparency and information about AI-enabled systems of care, and they also highlighted the importance of exercising agency over AI-enabled care. The proposed framework reflects the study’s findings by considering the proportionate degrees of information, agency, and risk posed by AI-enabled systems when determining the appropriate level of transparency. The framework itself also responds to the study’s findings that participants expressed a strong preference for standard protocols to guide the governance and implementation of AI-enabled systems. The study is solidly grounded in patient-centered care, which prioritizes individual values and preferences. The study and framework also reflect the current period of AI exceptionalism and the need to build patient trust in a relatively new and rapidly evolving technology.

## Supporting information

S1 Text Participant intake survey. This appendix includes all the components of the intake survey administered to participants, including both the demographic and Likert scale questions.
(DOCX)

S2 Text Interview script. This appendix includes all the components of the questions that were required to be asked during the interview, with the exact language that each interviewer used when asking said questions.
(DOCX)

S3 Text LLM coding protocol. This appendix documents the verbatim prompts and procedures used for the LLM-assisted qualitative thematic analysis described in Methods §2.4. The protocol was implemented as a four-module pipeline. Each module’s prompt is reproduced below as a template; placeholders enclosed in square brackets (e.g., [Insert Participant #]) were substituted with the relevant participant data when the prompt was executed.
(DOCX)

S1 Data Participant responses. This data includes the de-identified responses of participants to interview questions.
(XLSX)

S2 Data Minimal data set. This data includes the de-identified item-level intake-survey responses of all 21 respondents (rows in random order), aggregate demographic counts, and the values used to build Fig 1.
(XLSX)
