# Supplementary material for: Patient expectations for artificial intelligence transparency and agency: A qualitative study and proportionate framework for clinical artificial intelligence implementation
Source: PLOS Digit Health. 2026 Nov 4;5(11):e0001816. doi: 10.1371/journal.pdig.0001816 (PMC13734596; doi:10.1371/journal.pdig.0001816)
Supplement: Supplementary file 1 [file pdig.0001816.s001.docx]

Pre-Interview Intake Survey

**Demographic Questionnaire**

**Question 1:**What are your First and Last Names?

**Question 2:**What is your age?

**Question 3:** What is your identified Gender

- Male
- Female
- Other

**Question 4:** What is your identified race?

- American Indian or Alaska Native
- Asian
- Black or African American
- Native Hawaiian or Other Pacific Islander
- White

**Question 5:** What is your identified ethnicity?

- Hispanic or Latino
- Not Hispanic or Latino

**Question 6:** What is your county of residence (in New Jersey)?

**Question 7:** What is your highest level of education?

- Did not graduate from high school
- High School Diploma
- Associate’s Degree
- Bachelor’s Degree
- Graduate Degree
- Doctorate

**Likert Questions about Artificial Intelligence**

Please answer the following from Strongly Disagree, Disagree, Neutral, Agree, to Strongly Agree.

**Question 1:** I understand how artificial intelligence models are developed.

**Question 2:** I understand how artificial intelligence models work.

**Question 3:** Artificial intelligence has a role to play in the healthcare field.

**Question 4:** All patients should be informed about how artificial intelligence is used in their healthcare.

**Question 5:** Patients should have the option to opt out of artificial intelligence use in their healthcare.

**Question 6:** AI models should be given sole responsibility in making decisions on a patient’s treatment plan.

**Question 7:** The way artificial intelligence programs are utilized in healthcare today is acceptable in taking into account patient’s rights.
