# Supplementary material for: Patient expectations for artificial intelligence transparency and agency: A qualitative study and proportionate framework for clinical artificial intelligence implementation
Source: PLOS Digit Health. 2026 Nov 4;5(11):e0001816. doi: 10.1371/journal.pdig.0001816 (PMC13734596; doi:10.1371/journal.pdig.0001816)
Supplement: Supplementary file 2 [file pdig.0001816.s002.docx]

Interview Script: Vignettes and Questions

**Scenario 1**

John Doe is a 50-year-old male who came in to get his routine colonoscopy screening. The gastroenterologist uses an AI imaging technology to help identify potential lesions that should be considered for biopsy during the procedure.

**Question 1A:** Should John be informed that his doctor utilizes this technology?

During his colonoscopy, the AI system identifies a suspicious area; however, the gastroenterologist does not find the area significant and chooses not to get a biopsy.

**Question 1B:** Should John be notified that the doctor is disregarding an AI suggestion? Why or why not?

When informed about the use of AI imaging in his care, Mr. Doe exclaims that he “does not want private parts of his body to be videotaped for a computer to see.”

**Question 1C:** Does he have the right to deny the use of the AI imaging software in his colonoscopy?

**Question 1D:** What steps can be taken by the gastroenterologist’s office to better inform John about the use of AI in the care he would receive from this physician?

**Scenario 2**

John Smith is a 30-year-old male presenting to the Emergency Department after a fall on a hike where he broke his arm. A new AI mortality prediction model in the ED scans his chart and his current presentation, predicting that he has a 60% chance of dying within the next 12 months. The doctor is unsure of how the model came to this conclusion, and the model cannot explain to the doctor how it came to determine that risk.

**Question 2A:** Should the patient be informed about this prediction?

After further reviewing the chart the Doctor is not able to understand why the model predicted this, to him Mr. Smith appears to be a regular, healthy man. The doctor decides not to tell Mr. Smith about the prediction.

**Question 2B:** Is the Doctor wrong for not telling Mr. Smith?

The AI model’s prediction stays in the patient’s chart and is able to be viewed by his other physicians and the patient cannot get it removed.

**Question 2C:** Is there a way that this model could be better implemented?

**Question 2D:** Should the patient be able to object to it/remove it?

**Scenario 3**

A patient was admitted to the hospital due to recent fever and fatigue. In the emergency department, it was found that he had a bacterial infection in his blood and was admitted for IV antibiotic treatment. However, after many tests and cultures over the next few days, the medical team was still uncertain where the infection was coming from. The head physician then received a message from an AI model that was reviewing the patient’s case and recommended considering a bone infection that had gone unnoticed. The doctor then did a thorough physical exam and found the patient had a tender spot on his lower leg. He then got X-ray imaging, which confirmed a bone infection. After receiving the appropriate treatment, the patient improved and could be discharged home after a couple more days. The patient and his family praise the physician for figuring out the source of the infection.

**Question 3A:** Should the physician be required to inform the patient and family members that an AI model suggested his diagnosis?

The hospital administration recently decided to implement this new AI model to oversee all patients who were admitted.

**Question 3B:** Should the patient have been informed on admission that an AI model will have access to their medical information?

- If yes, how should this have been done?
- If no, why not?

**Non-Vignette Interview questions**

**Question 4:** What steps or actions by either a doctor or a clinic would help you trust AI use in your healthcare?

**Question 5:** Should a patient be informed of every use of AI in their healthcare?

**Question 6:** Doctors are not always experts in AI use, what in your opinion would be the best way for an individual to explain AI use to a patient?
