# Supplementary material for: Patient expectations for artificial intelligence transparency and agency: A qualitative study and proportionate framework for clinical artificial intelligence implementation
Source: PLOS Digit Health. 2026 Nov 4;5(11):e0001816. doi: 10.1371/journal.pdig.0001816 (PMC13734596; doi:10.1371/journal.pdig.0001816)
Supplement: Supplementary file 3 [file pdig.0001816.s003.docx]

# S3 Appendix. LLM Coding Protocol

This appendix documents the verbatim prompts and procedures used for the LLM-assisted qualitative thematic analysis described in Methods §2.4. The protocol was implemented as a four-module pipeline. Each module’s prompt is reproduced below as a template; placeholders enclosed in square brackets (e.g., [Insert Participant #]) were substituted with the relevant participant data when the prompt was executed.

## Model and generation settings (cross-reference to Methods §2.4)

- **Model:** Google’s Gemini 2.5 Pro Preview, model identifier gemini-2.5-pro-preview-06-05, released 2025-06-05
- **Access:** Google AI Studio
- **Top-p:** 0.95 (model default)
- **Top-k:** 64 (model default)
- **Temperature:** varied per module (specified below)
- **System instruction:** none beyond the role-framing embedded in each prompt

## Pipeline overview

The pipeline comprises four sequential modules. Modules 1–3 are responsible for the analytic operations summarized in Methods §2.4. Module 4 supported the selection of illustrative quotes presented in Results. Outputs from each module were reviewed by the research team before becoming inputs to the next module; per-module human review actions are described in Methods §2.4.

| Module | Operation | Temperature | Input | Output |
| --- | --- | --- | --- | --- |
| 1 | Open coding | 0.2 | One participant’s response to one question | List of codes, with quote and significance |
| 2 | Code consolidation and codebook construction | 0.3 | Master list of codes from Module 1 + question-level theme reference | Refined codes mapped to themes; emergent categories |
| 3 | Theme generation | 0.6 | Module 2 output | 4–7 overarching themes with definitions and contributing codes |
| 4 | Illustrative-quote extraction per theme | 0.6 | One theme from Module 3 + relevant participant response segments | Facets/insights per theme, each with attributed quotes |

Module 3 was executed multiple times to surface a range of plausible thematic structures; the research team selected the structure that best represented the data through structured discussion to interpretative consensus.

## Module 1 — Open Coding (per participant, per question)

**Operation.** For each participant’s response to each interview question, identify distinct statements expressing a perspective, concern, condition, suggestion, or reasoning, and assign a concise descriptive code to each.

**Temperature.** 0.2

**Input fields supplied per invocation:** - Participant ID - Question identifier and full question text - (Where applicable) the scenario text for scenario-based questions - The participant’s full response to that question - The overarching research goal (constant across invocations)

**Prompt template (verbatim):**

You are a qualitative researcher analyzing patient perspectives on AI in healthcare.
Your overall research goal is: "To determine patient perspectives on AI in healthcare."

Participant ID: [Insert Participant #]
Scenario (if applicable): [Paste Scenario text for Q1, Q2, or Q3]
Question Asked: [Insert Question ID, e.g., 1A]: "[Insert full text of Question 1A]"
Participant's Response:
"""
[Paste Participant's full response to this specific question]
"""

Your task is to:
1. Carefully read the participant's response to this specific question.
2. Identify distinct statements or segments (direct quotes) that express a clear perspective, opinion, concern, condition for acceptance, suggestion, or reasoning related to the question and AI in healthcare.
3. For each identified segment, generate a concise, descriptive code (a short phrase or keyword, typically 2-5 words) that captures the essence of that specific point.
4. Briefly explain (1 sentence) what this code signifies in terms of the patient's perspective on the question asked.

Present your output in the following format for EACH code found in this single response:
---
Code: [Your Generated Code]
Quote: "[Direct quote from the participant's response]"
Significance: [Your 1-sentence explanation of the perspective captured]
---

**Output.** Per invocation, a list of codes, each with the quote it derives from and a one-sentence explanation of its significance. All Module 1 outputs were compiled into a master list keyed by Participant ID and Question ID.

## Module 2 — Code Refinement and Initial Categorization

**Operation.** Consolidate the master list of Module 1 codes into a refined codebook by (a) merging conceptually similar codes, (b) mapping refined codes to pre-identified question-level themes where applicable, and (c) producing emergent categories for codes that did not fit existing themes.

**Temperature.** 0.3

**Input fields supplied per invocation:** - Master list of all codes from Module 1 (code, quote, significance, Participant ID, Question ID) - Pre-identified question-level themes (from the *Question Themes for Survey* reference table) - The overarching research goal (constant)

**Prompt template (verbatim):**

You are a qualitative researcher refining a large set of codes generated from patient interviews about AI in healthcare.
Your research goal is: "To determine patient perspectives on AI in healthcare."

You have a master list of initial codes (each with a quote, significance, participant ID, and question ID).
You also have a list of pre-identified themes associated with each survey question.

Your tasks are to:
1. Review all initial codes. Identify codes that are very similar in meaning. Suggest a 'Refined Code' for each group of similar codes (or keep original if unique). Provide a brief definition for each Refined Code.
2. For each Refined Code, try to map it to one of the pre-identified 'Survey Themes' for the question it originated from.
3. If a Refined Code (or group of codes) doesn't clearly fit an existing Survey Theme, or represents a new significant idea, group them into 'Emergent Categories' or 'Potential Sub-themes'. Provide a name and brief definition for these.
4. Indicate which Participant IDs and Question IDs contribute to each Refined Code or Emergent Category.

Present your output structured as follows:

**Refined Codes Mapped to Survey Themes:**
Survey Theme: [e.g., Transparency (from your CSV)] - Associated Question(s): [e.g., 1A, 1B, 1D, 2A, 3A, 5]
 - Refined Code 1: [Name] - Definition: [Brief definition] (Consolidates: [original_code_A, original_code_B]; From: P1/Q1A, P3/Q1A)
 - Refined Code 2: [Name] - Definition: [Brief definition] (From: P2/Q1B)
...

**Emergent Categories/Potential Sub-themes:**
Category Name 1: [Name] - Definition: [Brief definition]
 - Refined Code X: [Name] - Definition: [Brief definition] (From: P5/Q4, P7/Q6)
 - Refined Code Y: [Name] - Definition: [Brief definition] (From: P1/Q2C)
...

Input Data:
Master List of Initial Codes:
"""
[Paste your compiled list of Codes, Quotes, Significance, Participant ID, Question ID from Module 1]
"""
Pre-identified Survey Themes:
"""
[Paste content of question-level theme reference, e.g.,
Question 1A: Transparency
Question 1B: Transparency, Physician Judgement
...]
"""

**Output.** A structured codebook of refined codes mapped to question-level themes where applicable, plus emergent categories. The research team reviewed mappings for plausibility, adjusted theme boundaries, and finalized a consolidated codebook of 16 codes that became the input to Module 3.

## Module 3 — Theme Development

**Operation.** Generate 4–7 overarching themes that cut across questions and participants, each with a definition, a list of contributing refined codes, and a brief narrative summary.

**Temperature.** 0.6

**Repetition.** Module 3 was executed multiple times to surface alternative thematic structures. The research team reviewed the candidate structures and selected the structure that best represented the data through structured discussion to interpretative consensus.

**Input fields supplied per invocation:** - The refined codebook and emergent categories from Module 2 - The overarching research goal (constant)

**Prompt template (verbatim):**

You are a qualitative researcher developing overarching themes about patient perspectives on AI in healthcare.
Your research goal is: "To determine patient perspectives on AI in healthcare."

You have a structured list of refined codes, some mapped to initial survey themes per question, and some grouped into emergent categories.
Your task is to:
1. Analyze this entire structured list.
2. Identify and develop 4-7 major overarching themes that capture the most significant and recurrent patterns of patient perspectives regarding AI in healthcare. These themes should cut across different questions and scenarios where appropriate.
3. For each major theme:
 a. Provide a concise and descriptive Theme Name.
 b. Write a clear Definition of the theme (what core perspective or set of related perspectives does it represent?).
 c. List the key Refined Codes and/or Emergent Categories (from Module 2 output) that primarily contribute to this overarching theme.
 d. Briefly explain the overall narrative or story this theme tells about patient perspectives.

Present your output as follows:
Major Theme 1: [Theme Name]
 Definition: [Theme definition]
 Key Contributing Refined Codes/Categories:
 - [Refined Code/Category Name 1]
 - [Refined Code/Category Name 2]
 Narrative Summary: [Brief summary of what this theme signifies]

Major Theme 2: [Theme Name]
...

Input - Categorized Codes and Themes:
"""
[Paste the output from Module 2]
"""

**Output.** Per invocation, a list of 4–7 candidate themes with name, definition, contributing codes, and narrative summary. The final four themes reported in the paper (1. Ethical Prerequisites for AI Integration; 2. Systemic Prerequisites for Trust; 3. Preserving Clinician Oversight; 4. Strategies for Meaningful Communication) were selected and refined by the research team from the cross-invocation outputs.

## Module 4 — Illustrative-Quote Extraction per Major Theme

**Operation.** For each finalized theme from Module 3, identify 3–5 distinct facets or sub-points of the theme and select 1–3 supporting quotes per facet from the original participant responses.

**Temperature.** 0.6

**Input fields supplied per invocation:** - One Major Theme (name, definition) - Original participant response segments traced back from theme → contributing codes → original quotes - The overarching research goal (constant)

**Prompt template (verbatim):**

You are a qualitative researcher detailing the nuances of a major theme concerning patient perspectives on AI in healthcare.
Overall Research Goal: "To determine patient perspectives on AI in healthcare."
Current Major Theme: "[Insert Major Theme Name Here]"
Theme Definition: "[Insert Major Theme Definition Here]"

Below are segments of original participant responses that have been identified as relevant to this Major Theme.
Your task is to:
1. Review these relevant response segments.
2. Identify 3-5 distinct facets, nuanced arguments, specific conditions, or key insights that further explain and elaborate on this Major Theme.
3. For each facet/insight, select 1-3 of the most illustrative, concise, and impactful direct quotes from the provided responses that best exemplify it.

Present your output as follows:
Major Theme: [Theme Name]

Facet/Insight 1: [Concise statement of the facet/insight]
 - Illustrative Quote (Participant [ID], Question [ID]): "[Quote]"
 - Illustrative Quote (Participant [ID], Question [ID]): "[Quote]"

Facet/Insight 2: [Concise statement of the facet/insight]
 - Illustrative Quote (Participant [ID], Question [ID]): "[Quote]"
...

Relevant Original Participant Response Segments for this Theme:
"""
Participant [ID] - Question [ID]: "[Relevant quote or full response segment]"
Participant [ID] - Question [ID]: "[Relevant quote or full response segment]"
...
"""

**Output.** Per invocation, a list of facets per theme with attributed illustrative quotes. The research team reviewed each facet for distinctness and depth, and verified that selected quotes were accurately attributed and representative.

## Human review actions per module

The human-in-the-loop validation actions described in Methods §2.4 were:

- **After Module 1:** Verify that each code accurately captures the perspective expressed in the quote and significance statement. Identify response segments where additional codes were warranted and supplement.
- **After Module 2:** Verify the plausibility of merged-code groupings and the appropriateness of mappings to question-level themes. Rename, merge, or split refined codes and emergent categories as needed.
- **After Module 3 (repeated invocations):** Compare candidate thematic structures across invocations. Select the structure that best represents the data. Refine theme names, definitions, and narrative summaries.
- **After Module 4:** Verify facet distinctness, depth, and quote attribution.

## Inter-rater reliability subsample

3 randomly selected transcripts (15% of the corpus) were independently coded de novo by 3 research team members blinded to all Module 1 outputs from the corresponding transcripts. Codes were aligned at the segment level by paired adjudication using a standard code-alignment procedure. Cohen's κ was computed at the code-presence level (48 decisions per rater:

16 codes × 3 transcripts) for all six coder pairs:

**Coder pair	Cohen's κ	95% CI*	Landis & Koch**

A.R.U. vs D.B. 0.497	[0.333, 0.818]	Moderate

A.R.U. vs G.S. 0.231	[0.186, 0.347]	Fair

D.B. vs G.S. 0.405	[0.231, 0.538]	Fair

A.R.U. vs LLM	0.269	[0.045, 0.458]	Fair

D.B. vs LLM	0.437	[0.333, 0.522]	Moderate

G.S. vs LLM	0.304	[0.213, 0.412]	Fair

Mean human–human κ = 0.378 (range 0.231–0.497); mean human–LLM κ = 0.337 (range 0.269–0.437). *95% CIs from transcript-level bootstrap (1,000 iterations, seed 42). The human-LLM interval reported in Methods §2.4[0.045, 0.522] is the envelope of the three human-LLM pairwise CIs.
