# Supplementary material for: Patient expectations for artificial intelligence transparency and agency: A qualitative study and proportionate framework for clinical artificial intelligence implementation
Source: PLOS Digit Health. 2026 Nov 4;5(11):e0001816. doi: 10.1371/journal.pdig.0001816 (PMC13734596; doi:10.1371/journal.pdig.0001816)
Supplement: Supplementary file 4 [file pdig.0001816.s007.docx]

Dear Editors of PLoS Digital Health,

Thank you for the opportunity to submit a revised version of our manuscript, “Patient Expectations for Artificial Intelligence Transparency and Agency,” for consideration for publication in PLOS Digital Health. We are grateful to the editors and reviewers for their thoughtful and constructive feedback, which has helped us strengthen the manuscript. We have addressed each comment in turn below. For comments that resulted in revisions to the manuscript, we provide the relevant revised text with changes indicated. For comments where we have retained our original approach, we provide a detailed explanation of our rationale.

1. We note that this data set consists of interview transcripts. Can you please confirm that all participants gave consent for interview transcript to be published?

***Author Response:***

*Participants did NOT consent to publication of full interview transcripts. The IRB-approved Information Sheet (V2) authorized: (a) recording and transcription of the Google Meets interview, (b) storage of recordings and transcripts on secure Hackensack Meridian Health (HMH) servers, and (c) redaction of identifying information. It did NOT authorize public release of transcripts. We are therefore providing the de-identified participant responses as appendix 4 under restricted access as permitted by PLOS’s policy for sensitive participant data.*

*Restricted-access data statement:*

*The minimal dataset underlying the findings of this study consists of de-identified interview transcripts and a coded codebook. Because participants did not consent to the public release of full interview transcripts, these data cannot be deposited in an open repository.*

*1. Complete description of the dataset: De-identified transcripts of 20 semi-structured interviews with Patient and Family Advisory Council members across nine hospital sites in a large U.S. healthcare network, together with the final codebook, master code-quote map, and intake survey responses. All identifying information has been removed in accordance with HMH IRB-approved redaction procedures.*

*2. Nature of the restriction: Ethical - the IRB-approved participatory information documentation did not authorize public release of full transcripts; only redacted illustrative quotes were authorized for publication.*

*3. Body imposing the restriction: Hackensack Meridian Health Institutional Review Board (HMH IRB Protocol Pro2024-0296, “Building Trust in AI: A Patient Perspective on the Inclusion of AI in Healthcare,” Principal Investigator Charles E. Binkley, approved 18 November 2024).*

*4. Special access privileges: None. The authors received access to these data through ordinary IRB-approved research procedures available to any HMH-affiliated investigator with appropriate IRB approval.*

*5. Non-author institutional contact for data-access requests: Research Integrity Office, Hackensack Meridian Health Institutional Review Board. Email:* [*HMHIRB@hmhn.org*](mailto:HMHIRB@hmhn.org)*. Phone: 201-880-3669.*

**Reviewer #1**: The authors frame this work as “participatory AI” where “stakeholders have a meaningful say in building the AI models.” However, the study only elicited opinions from PFAC members post-hoc, with no evidence that participants influenced model design, deployment criteria, or governance. This is consultation, not participation. So, I propose that to rename “participatory” to “consultative” throughout. Acknowledgement as a limitation in Discussion. Remove claims about “building AI models together.”

***Author Response:***

*We agree. The methodological reality of the study is consultation, since we are eliciting and analyzing PFAC members' perspectives post hoc, which is not participation in the technical sense of co-design or shared governance. We agree that using “participatory” risks overstating both the methodology and the contribution. We have made three changes:*

*1. Terminology. Every methodological use of “participatory” has been replaced with “consultative” — specifically at the Author Summary and Introduction, the two locations in the original manuscript that characterized the present study as participatory. “Participation” has been retained where it refers exclusively to study enrollment (“participants in this study”), in accordance with the reviewer’s note. One unchanged occurrence remains in the References list, where “Participatory” appears in the title of a cited work (Delgado et al., The Participatory Turn in AI Design).*

*2. Removed overclaims. Language framing the study as one in which patients “build AI models” or share decision-making authority over model architecture, deployment, or governance has been removed. The revised Author Summary positions this work as gathering perspectives that “could inform future participatory AI development” rather than implementing such development.*

*3. New limitations addition. The Limitations subsection now contains a dedicated section that explicitly distinguishes consultation from participation and identifies the participatory governance structures the field still requires. The addition to the limitations section reads as follows:

“A further limitation concerns the nature of stakeholder engagement in this work. PFAC members were consulted to elicit their opinions, but they did not influence the design, deployment criteria, or governance of any AI model. This study should therefore be understood as a consultative rather than a fully participatory effort, and future work should engage stakeholders more directly in model development, deployment decisions, and oversight.”*

The proposed three-tier model (disclosure/assent/consent) mirrors decades-old clinical risk stratification. The authors do not differentiate AI-specific risks (e.g., black-box opacity, automation bias, model drift) from general procedural risks. The framework adds no new conceptual machinery. Specify which AI-specific attributes shift a system from “assent” to “consent” (e.g., non-explainability, lack of clinician override, proprietary data lock-in). Without this, the framework is generic.

***Author Response:***

*We agree that the original manuscript did not sufficiently articulate the AI-specific properties that govern tier assignment, and we have revised the Discussion to address this gap directly. We note, however, that the framework intentionally preserves flexibility in its tier descriptions to reflect the reality that individual health systems and providers will weigh these properties differently based on their own risk tolerance and clinical context, and may therefore reasonably arrive at different tier designations for the same system. That said, we recognize the original manuscript did not adequately specify the AI-specific attributes that inform this calculus, and the revised text now does so explicitly.*

*In the revised manuscript, we now specify several AI-specific attributes that can independently elevate a system's tier designation. First, we address non-explainability while also acknowledging the well-documented tension between interpretability and model performance. We argue that reliability within a defined clinical context is a more operationally appropriate measure than explainability per se, drawing an analogy to established clinical tools such as CT imaging, whose outputs clinicians rely upon without a mechanistic understanding. Under this logic, low-reliability systems would require consent-level engagement regardless of their explainability, while high-reliability systems may warrant only assent even in settings of elevated clinical risk.*

*Second, we now explicitly address clinician oversight and system autonomy as independent risk identifiers. Fully autonomous systems, as well as those that easily lead clinicians to automation bias that functionally reduce clinician engagement, are designated as requiring consent-level obligations, directly responding to the reviewer's concern about the lack of clinician override as a tier-shifting attribute.*

*Third, we identify proprietary data retention and restricted patient access to AI-generated outputs as factors that can elevate an otherwise low-risk tool to assent-level status, grounded in our participants' strongly expressed preferences regarding data privacy and the ability to contest medical record entries.*

*Collectively, these additions constitute the AI-specific conceptual machinery the reviewer identified as absent. The revised framework makes clear that tier assignment is governed not solely by the magnitude of clinical risk, but by the explainability, controllability, and data governance properties of each AI-enabled system. In addition to the expansion in the discussion, Table 2 has been updated to reflect the AI-specific conceptual machinery used in defining disclosure, assent, and consent.*

The authors claim “transparency is an ethical floor necessary for autonomy” and then use patient preferences to justify that floor. This is logically circular — patient preferences cannot ground an ethical principle without independent justification. The manuscript conflates empirical findings (patients want X) with normative claims (X is required ethically). Separate empirical from normative arguments. State clearly: “Patients believe transparency is necessary” (descriptive) vs. “We argue transparency is ethically required” (normative), and provide independent ethical reasoning (e.g., Beauchamp & Childress).

***Author Response:***

*We acknowledge that the original phrasing did not make this distinction explicit. The normative basis for transparency is grounded in the bioethical principle of respect for autonomy. Autonomy requires access to relevant information, and our empirical findings play a distinct and complementary role: they illustrate how patients recognize and expect that principle to be operationalized in the specific context of clinical AI. In this sense, the descriptive finding that patients want transparency and the normative claim that transparency is ethically required are not circular; the former confirms and contextualizes the latter without serving as its logical foundation.*

*We have revised the manuscript to make this distinction clearer. In the Discussion, we have added the following: "These findings echo the bioethical principle of respect for autonomy, demonstrating that transparency is not merely what patients prefer but the mechanism that makes autonomous decision-making in healthcare possible." This framing anchors the normative claim in established bioethical theory while preserving the study's empirical contribution.*

Dear Authors,
I believe the following two articles would strengthen your manuscript in distinct, non-overlapping ways. Adding them would significantly enhance the manuscript's theoretical depth and empirical grounding. Thank you for your consideration, and I look forward to seeing these important connections made visible in a revised version.

Citation #1
Precise placement:
After the sentence ending on Page 13 and 14: "As a bridge, clinicians are entrusted by their patients to interpret AI outputs, validate recommendations, and serve as the final arbiter of care decisions."
Exact quoted comment to be inserted as a citation:
"Implicit leadership theories (ILTs) shape how individuals evaluate, trust, and accept authority figures' decisions, particularly in hierarchical systems such as healthcare. When new technologies like AI are introduced, patients' pre-existing ILTs about clinicians as trustworthy 'bridges' may moderate their willingness to delegate oversight or accept algorithmic recommendations."
Reference: Amini S, Rahati M, Atash-Parvar S, Panahi M, Shahpari M. Implicit Leadership Theories and Traits: A Qualitative Study of Managers in a Medical Sciences University. J Healthc Leadersh. 2025;17:549-563.

***Author Response:***

*The clinician-as-bridge construct that emerged in our analysis (Theme 3) is consistent with the broader literature on implicit leadership theories (ILTs), which describe how individuals in hierarchical systems pre-formulate expectations about authority figures and use those expectations to evaluate decisions, including decisions to delegate trust to novel technologies. We have added Amini et al. (2025) at the location proposed by the reviewer, with a sentence that ties our finding to the ILT framework as one explanatory mechanism for why patients position the clinician, rather than the AI or the institution, as the locus of trustworthy mediation. The inserted sentence reads:*

*“This positioning of the clinician as a trusted intermediary is consistent with implicit leadership theories (ILTs), which describe how patients carry pre-formulated expectations of clinicians as authoritative interpreters and may use these expectations to determine whether to extend trust to new technologies introduced into clinical encounters.”*

Citation #2

Precise placement:
After the sentence ending on Page 16, lines 8–10:
"Our findings indicate that participants do not inherently grant trust to AI; rather, trust must be earned through evidence of systemic reliability. This includes standardized protocols, rigorous validation of AI models, and staff training."
Exact quoted comment to be inserted as a citation:
"Public health crises such as COVID-19 have demonstrated that systemic trust in healthcare infrastructure—rather than trust in any single technology—is the primary driver of patient adherence and acceptance of novel interventions. Health systems that fail to establish transparent, reliable safety protocols at the macro level undermine patient willingness to accept even low-risk automated tools."
Reference: Koolivand A, Amini S. *The importance of PHC-based measures in controlling COVID-19 outbreak: Implications for global health system.* Ethics, Medicine, and Public Health. 2020 Jun 17;14:100549.

***Author Response:***

*We have added Koolivand & Amini (2020) at the location proposed by the reviewer, with a sentence situating our findings on systemic trust within the broader public health literature on health-system reliability. The COVID-19 experience demonstrated empirically that public adherence to novel interventions depends not only on the intervention itself but on the perceived reliability of the broader health-system infrastructure delivering it. This finding strengthens our interpretation that AI adoption requires institutional rather than merely technological trust-building. The inserted sentence on page 14 reads:*

*“This pattern parallels findings from the public-health response to COVID-19, in which systemic trust in healthcare infrastructure, rather than confidence in any single tool, was the primary determinant of public adherence to novel interventions. AI systems introduced into low-trust institutional environments are unlikely to be accepted by patients regardless of the technology’s intrinsic reliability.”*

**Reviewer #2**: The authors used “Google’s Gemini 2.5 Pro for initial coding” but provide no prompt, no version lock, no temperature setting, no validation against human-only coding, and no inter-rater reliability with the LLM. “Rigorous human validation” is asserted without metrics.

***Author Response:***

*We thank Reviewer #2 for this important methodological critique, and we agree that the original Methods section did not provide the detail necessary for reproducibility or rigorous assessment of the LLM-assisted coding process. We have expanded §2.4 (“Data Analysis”) substantially to disclose:*

*1. Model version snapshot. The model used for all LLM-assisted coding reported in this paper was Gemini-2.5 Pro-preview-06-05 (Gemini 2.5 Pro Preview, released 2025-06-05), accessed via Google AI Studio.*

*2. Generation settings. Top-p was left at the model default of 0.95; top-k was left at the model default of 64. Temperature was deliberately varied across the three analysis modules to balance analytical consistency with the semantic breadth required for exploratory theme generation: 0.2 for open coding (Module 1), 0.3 for code consolidation (Module 2), and 0.6 for theme generation (Module 3), with Module 3 repeated multiple times to surface the range of plausible thematic structures before researcher selection and refinement.*

*3. Prompts. Verbatim prompts for each of the three analysis modules (per-response coding, code consolidation, theme generation), drawn from the Refined Modular Prompting Architecture used during analysis. These are included in a new supplement (S3 Appendix: LLM Coding Protocol).*

*4. Human validation procedure (primary analysis). A description of the iterative team-discussion process by which LLM outputs from each module were reviewed and refined to interpretative consensus, distinguished from the new inter-rater reliability sub-study (item 5 below).*

*5. Inter-rater reliability sub-study, framed within Braun & Clarke's reflexive thematic analysis paradigm. We respectfully note that reflexive thematic analysis (RTA), the framework guiding our analysis since the original submission, explicitly does not endorse inter-rater reliability statistics as quality criteria. Braun & Clarke (2019) argue that IRR contradicts the epistemological foundation of RTA, in which knowledge is treated as subjective and multiple researchers engage in negotiated meaning-making rather than tested classification. Braun & Clarke (2021) further argue that "coding reliability measures" are inappropriately promoted as universal requirements across paradigmatically distinct thematic-analysis approaches. The validation appropriate to RTA (methodological transparency, iterative team consensus, and reflexive engagement with the data) is precisely what we provide in the expanded §2.4 and S3 Appendix.*

*Nevertheless, in the spirit of the reviewer's request for transparency about LLM-assisted coding, we conducted a retrospective Cohen's κ sub-study on a randomly selected subsample of three transcripts (Interviews 1, 4, 9; 15% of the corpus; random seed 42). Three members of the research team (D.B., A.R.U., G.S.) independently coded these transcripts, blinded to the LLM's outputs.*

*Results:*

*- Mean human-vs-LLM κ = 0.337 (range 0.269–0.437; 95% CI from transcript-level bootstrap = [0.045, 0.522])*

*- Mean human-vs-human κ = 0.378 (range 0.231–0.497)*

*- Both indicate fair agreement per Landis & Koch (1977).*

*Two observations are relevant. First, the LLM's agreement with humans (0.337) is essentially equivalent to humans' agreement with each other (0.378), indicating that the LLM operated at the consistency level of an independent human coder on this subsample, which was not noticeably less reliable. Second, the modest-rather-than-substantial agreement across all pairs is consistent with the broader RTA literature on retrospective code-presence judgments by independent raters in interpretive paradigms (Braun & Clarke, 2019, 2021), and substantiates our methodological choice to use iterative team-discussion consensus rather than independent parallel coding for the primary analysis. We have added Braun & Clarke (2019, 2021) to the references. Per-pair κ values are reported in S3 Appendix.*

Participants are 95% White/non-Hispanic and 71% with bachelor’s degree or higher. This does not reflect general U.S. patient populations. The authors acknowledge this only in passing, yet the framework is presented as universally applicable. So Add a “Limitations” subsection in Discussion explicitly stating that findings may not generalize to non-White, lower-education, or rural populations. Recommend oversampling in future work.

***Author Response:***

*We agree. The submitted manuscript noted the sample's demographic composition but did not adequately address its implications for the framework’s scope. This has been addressed in the limitations subsection of the discussion, which explicitly addresses generalizability and recommends generalizable oversampling in future consultative and participatory work. However, this skew was an expected limitation in our study design, as we wanted to consult with a subpopulation of the general population with experience in patient advocacy within a large healthcare network. We planned to apply the resulting framework from this study to a more representative cohort in subsequent studies. The paragraph that addresses this is as follows:

“While this study provides a patient-centered framework for clinical AI implementation, it comes with its own limitations. The participant cohort was predominantly female, caucasian, and highly educated, which makes this sample unlikely to be generalizable to the general public. However, this skew was an expected limitation in our study design, as we intentionally sought out members of PFACs who serve as “key informants” that may possess unique insights given their experience in patient advocacy within a healthcare system. Engaging with this specialized population allowed us to establish a robust baseline for an ideal transparency framework. Nonetheless, we recognize that perceptions of AI, data privacy, and trust in medical institutions are heavily influenced by cultural, socioeconomic, and geographic factors. In the spirit of consultative AI, future studies are planned to build on this baseline framework and evaluate it in a broader, more generalizable patient population. This will ensure the final operational frameworks are adapted to protect and serve all populations. A further limitation concerns the nature of stakeholder engagement in this work. PFAC members were consulted to elicit their opinions, but they did not influence the design, deployment criteria, or governance of any AI model. This study should therefore be understood as a consultative rather than a fully participatory effort, and future work should engage stakeholders more directly in model development, deployment decisions, and oversight.”*

The framework uses “low/intermediate/high risk” but never defines what kind of risk (clinical, privacy, psychological, autonomy-related). An ambient scribe is called “intermediate risk” — based on what evidence? Provide an explicit risk typology (e.g., risk of misdiagnosis, risk of data breach, risk of patient distress). Justify each example’s tier with cited evidence or reasoned argument.

***Author Response:***

*We acknowledge that the original manuscript did not explicitly characterize the dimensions of risk relevant to AI-enabled systems. Our data affirm what has been previously reported in the literature on the value patients place on transparency about AI and align with published definitions of risk, such as those outlined by Rose & Shapiro (CHEST, 2024), which are already cited in our manuscript. We have added the following language to the framework description to make this point clearer:*

*"Risk in AI-enabled systems is multidimensional, encompassing factors such as the degree of model autonomy, the extent to which the system departs from established standards of care, whether the AI is patient-facing, and the direct clinical risk introduced by the system within its care context. Rather than prescribing a fixed risk typology, this framework is intentionally designed to be generalizable, recognizing that health systems must evaluate these dimensions within their own governance structures and local ecosystems."*

*Second, we have expanded the discussion of the ambient scribe example to provide explicit evidentiary justification for its intermediate risk classification. The revised passage now reads:*

*“Assent would be appropriately applied to ambient AI scribes, as there is an intermediate risk of harm, but a full informed consent discussion is not warranted. This is because passive audio capture in sensitive clinical encounters introduces data privacy vulnerabilities, and transcription inaccuracies can result in documentation errors with downstream clinical consequences. Our data showed that participants independently raised concerns about medical record accuracy and the permanence of erroneous data, lending direct empirical support to this classification. Furthermore, according to Rose & Shapiro's criteria, ambient scribes are patient-facing, present during the clinical encounter, and represent a meaningful departure from traditional documentation practices. These factors would collectively demonstrate an intermediate level of risk rather than a high one and support placement in the assent tier rather than simple disclosure."*

Patients want clinicians as “bridge” between AI and patient. But the manuscript does not specify what happens when clinician disagrees with AI, or when AI recommendations are opaque even to the clinician. Add a scenario analysis (e.g., “If clinician overrides AI, should patient be told?”). Discuss whether clinicians must be trained in AI explanation methods (e.g., LIME, SHAP).

***Author Response:***

*We thank the reviewer for raising a concern regarding what may happen when clinicians disagree with AI. In fact, in the scenarios our participants were given during the interviews, this exact scenario was introduced (Scenario 1, Question 1B), as provided in the S2 Appendix. Before the introduction of AI, clinicians had to adjudicate between contradictory and conflicting data on a regular basis, which patients rely on and trust their provider’s clinical judgment on the data given to them to develop the best treatment plan for their patients. While patients said they view their physicians as a “bridge” to AI, they ultimately agreed to trust their physicians' clinical judgment for final decisions about their care. Because of the findings in our data, we did not think it is necessary to conduct a separate scenario analysis since patient perspectives on the scenario of AI disagreement is already captured in our initial data from the interview, and that data shows that as long as clinicians are the final decision makers of patient’s treatment plans, the intricacies of how clinicians use AI input does not need to be disclosed to them. We addressed this in the following paragraph in the discussion as follows:*

*“This trust also has a practical implication for how clinician-AI disagreement should be handled: because patients have already delegated final judgment to their physician, a clinician overriding or discounting an AI recommendation is not a transparency failure requiring disclosure; it is precisely the oversight patients expect. Just as clinicians routinely adjudicate between conflicting data sources (discordant imaging and laboratory results, contradictory specialist opinions, etc.) without itemized explanation, AI output is best understood as another input absorbed into existing clinical judgment rather than as a parallel decision-making authority that must be publicly reconciled. From our data, it was clear that transparency between clinicians and their patients regarding AI involvement in their care should be known; however, the intricacies of how clinicians use AI input to make final clinical decisions, for example, disagreement with AI output and the clinician, do not require disclosure.”*

Theme 3 states clinicians must be the "final arbiter." Theme 1 states that patients must have full disclosure and agency. The manuscript never addresses what happens when a clinician, exercising judgment, decides not to disclose AI use because they believe it will cause unnecessary anxiety or confusion. Whose view prevails? Add a dedicated subsection in Discussion titled "Tensions between clinician oversight and patient transparency." Propose a resolution pathway (e.g., mandatory disclosure of AI use in decision-support, but optional disclosure for administrative AI).

***Author Response:***

*We thank the reviewer for identifying this apparent conflict and for the opportunity to clarify the boundaries of our findings. While we understand how Theme 1 (disclosure/agency) and Theme 3 (clinician as final arbiter) might appear to be in tension, our empirical data demonstrate that, from the patient's perspective, which is the explicit scope of this study, these two values are not in conflict.*

*In our semi-structured interviews, this exact operational nuance was actively explored. Specifically, in Question 5 of our interview guide (provided in the S2 Appendix), participants were asked directly whether every instance and use of AI must be disclosed to the patient, or if a clinician's judgment regarding the relevance of disclosure should apply.*

*Our data revealed that patients do not view full transparency and physician oversight as competing values that require a "winner" or a complex resolution pathway. Instead, patients inherently reconcile them: they desire overarching transparency about AI involvement in their care when it is materially important to their health outcomes, yet they simultaneously trust and expect their physicians to exercise clinical judgment regarding when and how to contextualize that information. Because patients explicitly delegate final decision-making authority to the clinician, they view the physician's choice of when to discuss an AI input as part of standard clinical judgment, rather than a failure of transparency.*

*Because this dynamic is already naturally balanced within the patient perspectives captured in our results, we do not believe there is an empirical basis in our data to declare an internal "tension" or to manufacture an artificial, function-based resolution pathway. While other informatics or bioethical studies may independently investigate the optimal institutional mechanisms for balancing clinician autonomy against algorithmic transparency, mapping out those specific operational rules falls outside the scope of this patient-centered qualitative study.*

*To ensure this clarity is reflected in the text, we have expanded our Discussion section to explicitly address how patient trust in the clinician's judgment naturally harmonizes the desire for agency with the necessity of clinician oversight:

“Importantly, our findings suggest that patients do not experience an inherent tension between their desire for transparency and the necessity of clinician oversight. Rather, patients inherently harmonize these principles by delegating operational decision-making to the provider, expecting overarching transparency regarding AI's presence in their care while trusting the clinician’s judgment to determine when and how specific algorithmic inputs are relevant to discuss."*

Thank you for your continued consideration of our manuscript. We look forward to further communication.

Dr. Guy Stein

Dr. Abinav Udaiyar

David Bouslov

Dr. Howard Ross

Dr. Charles Binkley
