# Supplementary material for: Patient expectations for artificial intelligence transparency and agency: A qualitative study and proportionate framework for clinical artificial intelligence implementation
Source: PLOS Digit Health. 2026 Nov 4;5(11):e0001816. doi: 10.1371/journal.pdig.0001816 (PMC13734596; doi:10.1371/journal.pdig.0001816)
Supplement: Peer review history

# Peer review: Decision Letter 0

**DOI:** 10.1371/journal.pdig.0001816.r001

**Authors:** Avishek Choudhury, Saeed Amini

27 May 2026

Response to Reviewers Revised Manuscript with Track Changes Manuscript **Journal Requirements:**

1. We note that this data set consists of interview transcripts. Can you please confirm that all participants gave consent for interview transcript to be published?

If they DID provide consent for these transcripts to be published, please also confirm that the transcripts do not contain any potentially identifying information (or let us know if the participants consented to having their personal details published and made publicly available). We consider the following details to be identifying information:

- Names, nicknames, and initials

- Age more specific than round numbers

- GPS coordinates, physical addresses, IP addresses, email addresses

- Information in small sample sizes (e.g. 40 students from X class in X year at X university)

- Specific dates (e.g. visit dates, interview dates)

- ID numbers

Or, if the participants DID NOT provide consent for these transcripts to be published:

- Provide a de-identified version of the data or excerpts of interview responses

- Provide information regarding how these transcripts can be accessed by researchers who meet the criteria for access to confidential data, including:

a) the grounds for restriction

b) the name of the ethics committee, Institutional Review Board, or third-party organization that is imposing sharing restrictions on the data

c) a non-author, institutional point of contact that is able to field data access queries, in the interest of maintaining long-term data accessibility.

d) Any relevant data set names, URLs, DOIs, etc. that an independent researcher would need in order to request your minimal data set.

For further information on sharing data that contains sensitive participant information, please see: https://journals.plos.org/plosone/s/data-availability#loc-human-research-participant-data-and-other-sensitive-data

If there are ethical, legal, or third-party restrictions upon your dataset, you must provide all of the following details (https://journals.plos.org/plosone/s/data-availability#loc-acceptable-data-access-restrictions):

1. A complete description of the dataset

2. The nature of the restrictions upon the data (ethical, legal, or owned by a third party) and the reasoning behind them

3. The full name of the body imposing the restrictions upon your dataset (ethics committee, institution, data access committee, etc)

4. If the data are owned by a third party, confirmation of whether the authors received any special privileges in accessing the data that other researchers would not have

5. Direct, non-author contact information (preferably email) for the body imposing the restrictions upon the data, to which data access requests can be sent

2. We ask that a manuscript source file is provided at Revision. Please upload your manuscript file as a .doc, .docx, .rtf or .tex.

3. Please upload separate figure files in .tif or .eps format. Also, remove the figures from your manuscript file but keep the legends.

For more information about figure files please see our guidelines:

https://journals.plos.org/digitalhealth/s/figures

https://journals.plos.org/digitalhealth/s/figures#loc-file-requirements

**Additional Editor Comments (if provided):** **Reviewers' Comments:**

**Comments to the Author**

1. Does this manuscript meet PLOS Digital Health’s publication criteria?

Reviewer #1: Yes

Reviewer #2: Yes

**********

2. Has the statistical analysis been performed appropriately and rigorously?

Reviewer #1: Yes

Reviewer #2: Yes

**********

3. Have the authors made all data underlying the findings in their manuscript fully available (please refer to the Data Availability Statement at the start of the manuscript PDF file)?

The PLOS Data policy

Reviewer #1: Yes

Reviewer #2: Yes

**********

4. Is the manuscript presented in an intelligible fashion and written in standard English?

Reviewer #1: Yes

Reviewer #2: Yes

**********

**Reviewer #1** : The authors frame this work as “participatory AI” where “stakeholders have a meaningful say in building the AI models.” However, the study only elicited opinions from PFAC members post-hoc, with no evidence that participants influenced model design, deployment criteria, or governance. This is consultation, not participation. So, I propose that to rename “participatory” to “consultative” throughout. Acknowledge as a limitation in Discussion. Remove claims about “building AI models together.”

The proposed three-tier model (disclosure/assent/consent) mirrors decades-old clinical risk stratification. The authors do not differentiate AI-specific risks (e.g., black-box opacity, automation bias, model drift) from general procedural risks. The framework adds no new conceptual machinery. Specify which AI-specific attributes shift a system from “assent” to “consent” (e.g., non-explainability, lack of clinician override, proprietary data lock-in). Without this, the framework is generic.

The authors claim “transparency is an ethical floor necessary for autonomy” and then use patient preferences to justify that floor. This is logically circular — patient preferences cannot ground an ethical principle without independent justification. The manuscript conflates empirical findings (patients want X) with normative claims (X is required ethically). Separate empirical from normative arguments. State clearly: “Patients believe transparency is necessary” (descriptive) vs. “We argue transparency is ethically required” (normative), and provide independent ethical reasoning (e.g., Beauchamp & Childress).

Dear Authors,

I believe the following two articles would strengthen your manuscript in distinct, non-overlapping ways. Adding them would significantly enhance the manuscript's theoretical depth and empirical grounding. Thank you for your consideration, and I look forward to seeing these important connections made visible in a revised version.

Citation #1

Precise placement:

After the sentence ending on Page 13 and 14: "As a bridge, clinicians are entrusted by their patients to interpret AI outputs, validate recommendations, and serve as the final arbiter of care decisions."

Exact quoted comment to be inserted as a citation:

"Implicit leadership theories (ILTs) shape how individuals evaluate, trust, and accept authority figures' decisions, particularly in hierarchical systems such as healthcare. When new technologies like AI are introduced, patients' pre-existing ILTs about clinicians as trustworthy 'bridges' may moderate their willingness to delegate oversight or accept algorithmic recommendations."

Reference: Amini S, Rahati M, Atash-Parvar S, Panahi M, Shahpari M. Implicit Leadership Theories and Traits: A Qualitative Study of Managers in a Medical Sciences University. J Healthc Leadersh. 2025;17:549-563.

Citation #2

Precise placement:

After the sentence ending on Page 16, lines 8–10:

"Our findings indicate that participants do not inherently grant trust to AI; rather, trust must be earned through evidence of systemic reliability. This includes standardized protocols, rigorous validation of AI models, and staff training."

Exact quoted comment to be inserted as a citation:

"Public health crises such as COVID-19 have demonstrated that systemic trust in healthcare infrastructure—rather than trust in any single technology—is the primary driver of patient adherence and acceptance of novel interventions. Health systems that fail to establish transparent, reliable safety protocols at the macro level undermine patient willingness to accept even low-risk automated tools."

Reference: Koolivand A, Amini S. *The importance of PHC-based measures in controlling COVID-19 outbreak: Implications for global health system.* Ethics, Medicine, and Public Health. 2020 Jun 17;14:100549.

**Reviewer #2** : The authors used “Google’s Gemini 2.5 Pro for initial coding” but provide no prompt, no version lock, no temperature setting, no validation against human-only coding, and no inter-rater reliability with the LLM. “Rigorous human validation” is asserted without metrics.

Participants are 95% White/non-Hispanic and 71% with bachelor’s degree or higher. This does not reflect general U.S. patient populations. The authors acknowledge this only in passing, yet the framework is presented as universally applicable. So Add a “Limitations” subsection in Discussion explicitly stating that findings may not generalize to non-White, lower-education, or rural populations. Recommend oversampling in future work.

The framework uses “low/intermediate/high risk” but never defines what kind of risk (clinical, privacy, psychological, autonomy-related). An ambient scribe is called “intermediate risk” — based on what evidence? Provide an explicit risk typology (e.g., risk of misdiagnosis, risk of data breach, risk of patient distress). Justify each example’s tier with cited evidence or reasoned argument.

Patients want clinicians as “bridge” between AI and patient. But the manuscript does not specify what happens when clinician disagrees with AI, or when AI recommendations are opaque even to the clinician. Add a scenario analysis (e.g., “If clinician overrides AI, should patient be told?”). Discuss whether clinicians must be trained in AI explanation methods (e.g., LIME, SHAP).

Theme 3 states clinicians must be the "final arbiter." Theme 1 states patients must have full disclosure and agency. The manuscript never addresses what happens when a clinician, exercising judgment, decides not to disclose AI use because they believe it will cause unnecessary anxiety or confusion. Whose view prevails? Add a dedicated subsection in Discussion titled "Tensions between clinician oversight and patient transparency." Propose a resolution pathway (e.g., mandatory disclosure of AI use in decision-support, but optional disclosure for administrative AI).

**********

what does this mean?). If published, this will include your full peer review and any attached files.

**Do you want your identity to be public for this peer review?**  If you choose “no”, your identity will remain anonymous but your review may still be made public.

For information about this choice, including consent withdrawal, please see our Privacy Policy

Reviewer #1: No

Reviewer #2: No

**********

**Figure resubmission:**

**Reproducibility:**

To enhance the reproducibility of your results, we recommend that authors of applicable studies deposit laboratory protocols in protocols.io, where a protocol can be assigned its own identifier (DOI) such that it can be cited independently in the future. Additionally, PLOS ONE offers an option to publish peer-reviewed clinical study protocols. Read more information on sharing protocols at https://plos.org/protocols?utm_medium=editorial-email&utm_source=authorletters&utm_campaign=protocols

# Author comment: Author response to Decision Letter 1

**DOI:** 10.1371/journal.pdig.0001816.r002

23 Jun 2026

Attachment Submitted filename: Response to Reviewers.docx

# Editor report: Decision Letter 1

**DOI:** 10.1371/journal.pdig.0001816.r003

**Authors:** Baki Kocaballi, Saeed Amini

12 Oct 2026

Patient Expectations for Artificial Intelligence Transparency and Agency: A Qualitative Study and Proportionate Framework for Clinical Artificial Intelligence Implementation

PDIG-D-26-00549R1

Dear Dr. Stein,

We are pleased to inform you that your manuscript 'Patient Expectations for Artificial Intelligence Transparency and Agency: A Qualitative Study and Proportionate Framework for Clinical Artificial Intelligence Implementation' has been provisionally accepted for publication in PLOS Digital Health.

Before your manuscript can be formally accepted you will need to complete some formatting changes, which you will receive in a follow-up email from a member of our team.

Please note that your manuscript will not be scheduled for publication until you have made the required changes, so a swift response is appreciated.

IMPORTANT: The editorial review process is now complete. PLOS will only permit corrections to spelling, formatting or significant scientific errors from this point onwards. Requests for major changes, or any which affect the scientific understanding of your work, will cause delays to the publication date of your manuscript.

If your institution or institutions have a press office, please notify them about your upcoming paper to help maximize its impact. If they'll be preparing press materials, please inform our press team as soon as possible -- no later than 48 hours after receiving the formal acceptance. Your manuscript will remain under strict press embargo until 2 pm Eastern Time on the date of publication. For more information, please contact digitalhealth@plos.org.

Thank you again for supporting Open Access publishing; we are looking forward to publishing your work in PLOS Digital Health.

Best regards,

Saeed Amini

Academic Editor

PLOS Digital Health

***********************************************************

**Additional Editor Comments (if provided):**

**Reviewer Comments (if any, and for reference):**
